# Homocysteine as a Predictor of Paroxysmal Atrial Fibrillation-Related Events: A Scoping Review of the Literature

**DOI:** 10.3390/diagnostics12092192

**Published:** 2022-09-09

**Authors:** Panagiotis Charalampidis, Eleftherios Teperikidis, Aristi Boulmpou, Christodoulos E. Papadopoulos, Victoria Potoupni, Konstantina Tsioni, Pantelitsa Rakitzi, Theodoros Karamitsos, Vassilios Vassilikos

**Affiliations:** 1 Third Department of Cardiology, Ippokratio General Hospital, Aristotle University of Thessaloniki, 54642 Thessaloniki, Greece; 2 St. Luke’s Hospital, 55236 Thessaloniki, Greece; 3 Biopathology Laboratory, Ippokratio General Hospital, 54642 Thessaloniki, Greece; 4 First Department of Cardiology, AHEPA University Hospital, Aristotle University of Thessaloniki, 54621 Thessaloniki, Greece

**Keywords:** homocysteine, arrhythmia, atrial fibrillation, oxidative stress

## Abstract

High levels of homocysteine (Hcy) have been linked with adverse cardiovascular outcomes, such as arrhythmias and stroke. In the context of paroxysmal atrial fibrillation (PAF), hyperhomocysteinemia has been demonstrated to be an independent predictor of future events. The aim of this report was to address the potential value of Hcy levels in predicting future paroxysms of atrial fibrillation (AF), as well as to identify the potential mechanisms of action. We searched PubMed and the Cochrane Database on 16 January 2022. Keywords used were homocysteine or hyperhomocysteinemia paired with a total of 67 different keywords or phrases that have been implicated with the pathogenesis of AF. We included primary reports of clinical and non-clinical data in the English language, as well as systematic reviews with or without meta-analyses. We placed no time constraints on our search strategy, which yielded 3748 results. Following title review, 3293 reports were excluded and 455 reports were used for title and abstract review, after which 109 reports were finally used for full-text review. Our review indicates that Hcy levels seem to hold a predictive value in PAF. Herein, potential mechanisms of action are presented and special considerations are made for clinically relevant diagnostic procedures that could complement plasma levels in the prediction of future PAF events. Finally, gaps of evidence are identified and considerations for future clinical trial design are presented.

## 1. Introduction

Atrial fibrillation (AF) represents the most common cardiac rhythm disorder, affecting millions of individuals worldwide, while its prevalence is expected to grow alarmingly within the next 50 years [1]. In the context of the substantial burden that AF poses on global health, on health economics, and on the affected individuals’ health-related quality of life, scientists have been focusing on decoding the disease’s exact pathophysiology; one cannot doubt the fact that AF is a result of multiple underlying factors, while it is usually precipitated by unknown triggers [2]. Research has shown that inflammation, in all its forms, is strongly linked with AF development; circulating inflammatory factors in the context of systemic inflammatory responses lead to atrial remodeling and fibrosis, a state that serves as a preamble for AF [3]. From this perspective, the identification of potential inflammation markers may be of paramount importance for the optimal diagnosis and management of the disease.

Homocysteine (Hcy) is a non-proteinogenic amino acid that is synthesized by methionine. Hcy serves as a precursor to many different amino acids in a series of biochemical reactions that are catalyzed by B vitamins. Folic acid (vitamin B9) seems to play an essential role in the conversion of Hcy to cysteine. In fact, folic acid supplementation has been demonstrated to effectively reduce plasma levels of Hcy [4]. Furthermore, an association between elevated Hcy and the methylene tetrahydrofolate reductase (MTHFR) 677C-allele polymorphism (rs1801133) has been demonstrated [5]. High levels of Hcy have been associated with endothelial cell injury and subsequent blood vessel inflammation. This has been linked to atherogenesis and ischemia [6]. However, a true causative effect that could prove a connection between these two entities, has not been yet established [7].

Paroxysmal atrial fibrillation (PAF), a very common form of atrial fibrillation (AF), is defined as an arrhythmic event that occurs spontaneously and is terminated within 7 days of onset [8]. According to epidemiological data, PAF occurs in approximately 25% of patients with a history of AF, while it has been linked with equally high morbidity and mortality as in permanent AF [9,10]. At the same time, PAF is a condition that has been correlated to high levels of Hcy. In fact, a positive correlation between levels of Hcy and future paroxysms of AF has been demonstrated in several clinical trials and reports [11,12,13,14,15,16,17,18,19,20,21,22] (Table 1). Furthermore, 2 systematic reviews with meta-analyses of these trials confirmed a positive correlation between elevated Hcy levels and recurrence of PAF [23,24] (Table 1). 

Based on the above findings, we ought to perform a scoping review of available clinical and non-clinical data with the aim to identify reports that could shed light on the potential mechanisms responsible for this specific effect. Scoping reviews are designed to address broader questions as compared to systematic reviews. They still rely on the established systematic review process, however, whereas a systematic review would attempt to answer a specific question (i.e., can Hcy levels be used to predict future paroxysms of AF?), scoping reviews usually tackle multiple questions, in an attempt to provide an exhaustive look at the available evidence. The goal of our effort was to identify any correlation between Hcy and PAF, identify underlying mechanisms, assess the interplay with other biomarkers, identify gaps of evidence, and ultimately provide insight that could assist in the initial clinical assessment and in the development of the optimal therapeutic strategies for each patient with PAF.

## 2. Methods

This scoping review was conducted in accordance with the Preferred-Reporting-Items-for-SystematicReviews-and-Meta-Analyses (PRISMA) guidelines (Supplementary Table S1). A search of PubMed and the Cochrane database was performed on 16 January 2022, based on a prespecified search protocol. We generated keywords based on the known mechanisms of AF pathogenesis. More specifically, the search terms used were homocysteine or hyperhomocysteinemia paired with one of the following: atrial, fibrillation, atrial fibrillation, fibrosis, cardiac fibrosis, myocardial fibrosis, atrial structural remodeling, left atrial appendage thrombus, cardiac inflammation, cardiovascular inflammation, vascular inflammation, mitral stenosis, mitral regurgitation, tricuspid regurgitation, cardiovascular oxidative stress, cardiac oxidative stress, vascular oxidative stress, myosin heavy chains, sarcoidosis, renin, angiotensin, aldosterone, RAAS, matrix metalloproteinases, MMP, disintegrin, sinus node, atrioventricular node, sick sinus syndrome, pulmonary veins, cardiac action potential, refractory period, wavelength, multiple wavelet, re-entrant leading cycle, electrical spiral waves, rotors, calcium, potassium, sodium, L-type calcium channels, calcium sensitivity, intracellular calcium, inward rectifier potassium ion channels, vagal, parasympathetic, sympathetic, epinephrine, norepinephrine, adrenaline, adrenergic, beta-2 receptors, sarcoplasmic reticulum, vortex shedding, gap junction proteins, GJA1, GJA5, connexin, thyroid, thyroid stimulating hormone, hyperthyroidism, hypothyroidism, troponin, BNP, NT-pro-BNP, electrocardiogram, ECG. 

We included primary reports of clinical and non-clinical data in the English language, as well as systematic reviews with or without meta-analyses. Narrative reviews, expert opinions and other types of medical correspondence were excluded. We placed no time constraints on our search strategy. Two independent reviewers participated in the selection of included reports (PC and ET). If differences were reported in included reports, a resolution was achieved via discussion and refereeing by a third reviewer (CP). Data were extracted using a custom, previously tested form for non-clinical data, while a standard PICO form was used for clinical data. 

## 3. Results 

**Figure 1:**
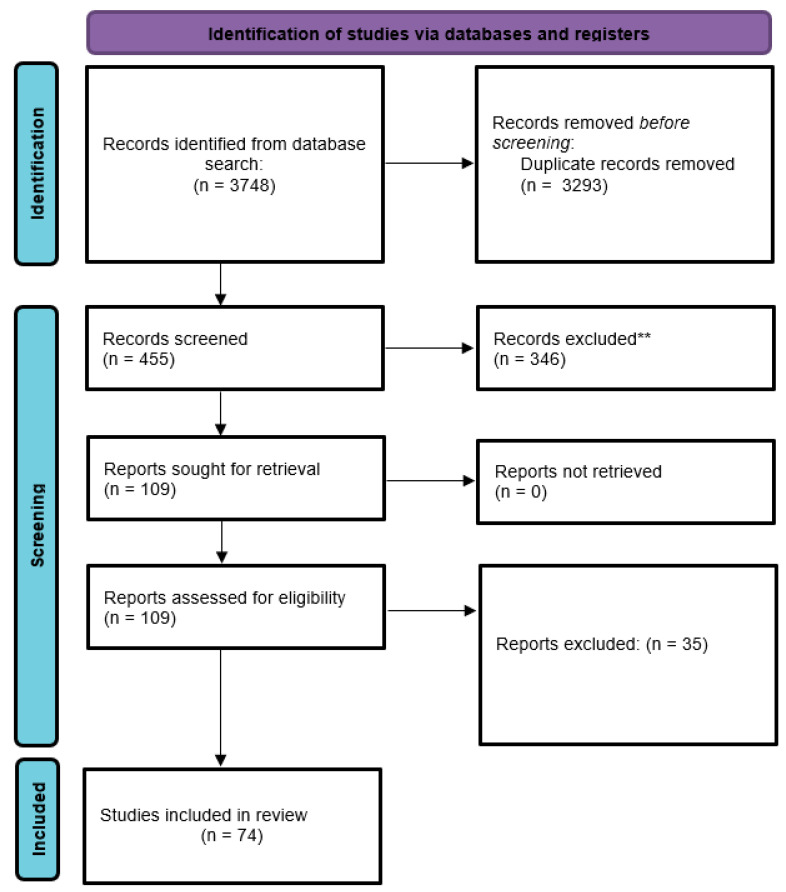
PRISMA flow diagram. From: Page MJ, McKenzie JE, Bossuyt PM, Boutron I, Hoffmann TC, Mulrow CD, et al. The PRISMA 2020 statement: an updated guideline for reporting systematic reviews. BMJ 2021; 372: n71. doi: 10.1136/bmj.n71. For more information, visit: http://www.prisma-statement.org/. Accessed on 28 January 2022.

Our search strategy yielded 3748 results. Following title review, 3293 reports were excluded and 455 reports were used for title and abstract review, after which 109 reports were selected for full-text review. We finally included 74 reports in our scoping review (Figure 1).

### 3.1. Non-Clinical Data

Our search strategy yielded 75 reports of in vitro and in animal models assessing the potential mechanisms of Hcy-induced direct myocardial toxicity. Table 2 provides a condensed overview of these reports. It should be noted that our search strategy did include neither the effects of Hcy on the vasculature nor data regarding its prothrombotic effects, both of which have been established in the literature and could contribute to the pathogenesis of cardiac disease. 

### 3.2. Oxidative Stress, Cardiac Fibrosis, and Remodeling

As the development of AF seems to be a result of the combination of a plethora of risk factors and comorbidities, oxidative stress has been associated with incident AF, while oxidative stress-induced atrial remodeling is considered the most common underlying mechanism; according to several reports, hyperhomocysteinemia has been linked with endothelial dysfunction, thus it represents an important factor for cardiovascular morbidity and mortality [93,94]. We were able to locate a total of 26 reports assessing oxidative stress as a potential mechanism of Hcy-induced cardiotoxicity [25,26,27,28,29,30,31,32,33,34,35,36,37,38,39,40,41,42,43,44,45,46,48,49,50,95]. Of these, 25 trials reported a positive correlation between Hcy and cardiomyocyte oxidative stress, while only one report failed to demonstrate oxidative stress as a cause of electron transport chain dysfunction, possibly due to an increased expression of the other protective mitochondrial proteins [41]. In these experiments, several different molecules were tested for their potential to inhibit the Hcy-induced oxidative stress, such as taurine and hydrogen sulfide. Taurine is an organic osmolyte involved in cell volume regulation, located in all organs throughout the body, which provides a substrate for the formation of bile salts; taurine seems to play a fundamental role in the modulation of intracellular free calcium concentration [96]. In parallel, hydrogen sulfide (H2S), the third discovered endogenous gas transmitter in mammals after NO and CO, participates in various pathophysiological processes; previous in vitro and in vivo research have revealed the protective role of H2S in the cardiovascular system that renders it useful in the protection of the myocardium against ischemia-reperfusion injury [97]. Interestingly, taurine [43,44] and hydrogen sulfide [45,46] were shown to be effective in counteracting a significant inhibiting effect against Hcy oxidative action. However, the clinical significance of that remains questionable, since none of the aforementioned agents used to hold a place in everyday clinical practice. 

Furthermore, we were able to locate 16 trials reporting cardiac fibrosis as a result of Hcy-induced oxidative stress [41,48,49,50,51,52,53,54,55,56,57,58,59,60,61,62]. Several mechanisms of action have been proposed for this effect, including increased reactive oxygen species, nitrotyrosine, matrix metalloproteinase, and decreased endothelial nitric oxide in response to antagonizing PPAR-gamma [82] and regulation of the Akt/FoxO3 pathway. More specifically, FoxO3 (forkhead box O3) is a member of the forkhead box transcription factors of the O class with a conserved helix-loop-helix DNA-binding domain; it is involved in a variety of vital cellular processes including oxidative stress, DNA repair, apoptosis, metabolism and cell cycle arrest [98,99]. Although the existing data are controversial, FoxO3 seem to play an important role in maintaining cardiovascular homeostasis. 

Finally, 11 trials were reported on cardiac remodeling because of hyperhomocysteinemia [60,61,62,63,65,66,67,68,69,70,71]. Again, several mechanisms of action are postulated, including increased expression of transforming growth factor-beta1 (TGFβ1), a significant increase in the ratio of collagenous to non-collagenous protein due to reactive interstitial fibrosis, and increased myocardial oxidative stress, increased expression of matrix metalloproteinase-2, matrix metalloproteinase-9 and decreased expression of connexin 40, 43 and 45, and suppression of the Nrf2/HO-1 pathway and Nrf2 nuclear transport. In fact, the activation of mitochondrial matrix metalloproteinase-9 (MMP9) can lead to cardiomyocyte dysfunction, in part, by inducing mitochondrial permeability (MPT). This has been demonstrated to be mediatethe d by binding of homocysteine to the N-methyl-d-aspartate receptor 1 (NMDA-R1) [86,87,88,89].

### 3.3. Electrical Remodeling

Electrical remodeling, a variety of changes in the electrical substrate of the heart capable of triggering AF onset, belongs among the most common risk factors for AF development [100]. We were able to locate 11 trials assessing the effect of Hcy on the electrical conductivity of the heart and an additional 4 trials reporting on alterations of beta 2 adrenergic receptors. This was demonstrated as a significant prolongation of QRS, QTc, and PR intervals on the electrocardiogram (ECG) [71,72,81]. The above point toward a potential effect of Hcy in practically all distinct phases of the heart’s systolic/diastolic cycle, demonstrating a clear arrhythmogenic potential associated with high levels of Hcy. Furthermore, Hcy has been reported to down-regulate the production of beta 2 adrenergic receptors [83,84], as well as to increase responsiveness to beta-adrenergic agonists [85]. Finally, alterations involving atrial calcium [74], sodium [77,78,80] and potassium [75,78,79,101] channels have also been demonstrated in hyperhomocysteinemia. 

## 4. Clinical Data

The available clinical data are highly heterogeneous. Furthermore, a positive correlation between Hcy levels and PAF has already been demonstrated in recent meta-ana-lyses. We, therefore, decided not to proceed with a meta-analysis. Available clinical evidence is presented narratively and in tabular format (Table 3). 

### 4.1. Oxidative Stress, Fibrosis, Thrombosis, and Remodeling

In a prospective cohort of 643 patients, the relationship between plasma Hcy and glutathione peroxidase (GPx)-1 levels was addressed. GPx-1 has been demonstrated to modulate cardiovascular risk related to Hcy via its antioxidant properties. After a median follow-up of 7.1 years, the authors reported an inverse relationship between Hcy and GPx-1 levels and the occurrence of cardiovascular events. In fact, patients with low GPx-1 and high Hcy levels were 3.2 times more likely to experience a cardiovascular event. The authors went on to recommend that GPx-1 levels are taken into consideration when Hcy levels are used to predict future cardiovascular events [102].

Overall, high levels of Hcy have been implicated in the induction of oxidative stress in the vasculature. While our search was not designed to locate such trials, the capacity of Hcy to induce oxidative stress with subsequent clot detachment which ultimately leads to ischemic stroke is well described in the literature [103]. In fact, patients with elevated Hcy levels have been demonstrated to have a 2.73-fold increased probability of ischemic stroke [104].

In a sample of 2697 patients from the Framingham Heart Study, left ventricular (LV) mass, wall thickness, and relative wall thickness were correlated to high Hcy levels in women but not in men. However, plasma Hcy was not related to left atrial size or LV fractional shortening in either sex [105].

In a study of 66 patients, elevated Hcy levels were linked with a disproportional LV dilatation, where the ensuing hypertrophy was not sufficient to compensate for the increased wall stress. The authors proposed that a potential mechanism is the hyperhomocysteinemia-associated increased oxidative stress which leads to the degradation of collagen, with consecutive fiber slippage and cardiac dilatation [106]. 

### 4.2. Electrical Remodeling 

In a population-based study of 7002 participants, 12-lead ECGs were performed and correlated to plasma Hcy levels. The authors demonstrated an association between the prolonged QTc interval and high Hcy levels [107]. Furthermore, in a retrospective database study, 178 patients were stratified according to QRS interval duration and plasma Hcy levels. The authors reported a significant correlation between higher levels of Hcy and longer QRS intervals. Other ECG parameters, such as PQ interval, QTc interval, and QT dispersion were not found to be statistically correlated to Hcy levels [108].

### 4.3. Relation to Left Ventricular Ejection Fraction and Other Biomarkers

In a prospective case-control study of 515 coronary artery disease patients and 194 controls, higher Hcy levels were correlated with reduced left ventricular ejection fraction (LVEF) (<40%). Furthermore, elevated Hcy levels were associated with increases in *N*-terminal pro-B-type natriuretic peptide (NT-pro-BNP) levels [109]. In another study of 1020 patients, high levels of Hcy were demonstrated to predict increased NT-pro-BNP (or BNP) levels [110]. NT-proBNP and Hcy levels were measured in 31 patients with type 2 diabetes mellitus. The authors reported a correlation between elevated NT-proBNP and Hcy levels in patients with LV diastolic dysfunction [111]. A trial of 227 patients demonstrated that the MTHFR C677T mutation which is associated with plasma Hcy levels, is also linked with plasma BNP levels, leading to the conclusion that plasma Hcy levels are positively correlated with plasma BNP levels [112].

In a community-based trial of 1497 patients, serum Hcy levels were associated with a higher likelihood of detectable high sensitivity troponin T (hs-cTnT). The effect was stronger among the elderly patients in the cohort (>65 years old), while no association was recorded in patients <65 years old. The authors concluded that the levels of serum Hcy are associated with hs-cTnT levels in the elderly, which could indicate a possible relationship between Hcy and subclinical myocardial damage [113]. In another trial of 194 consecutive patients with acute myocardial infarction, elevated serum Hcy levels were positively correlated with serum cardiac troponin-I [114].

In a prospective cohort of 1224 consecutive patients with subclinical AF (SCAF), Hcy and uric acid (UA) were significantly elevated. The authors reported that an increase of 1 standard deviation (SD) in Hcy (5.7 μmol/L) levels was associated with an increased risk of SCAF in men and women regardless of their UA levels. Similarly, a 1-SD increase in UA (91 μmol/L) was associated with an increased risk of SCAF among the patients with high levels of Hcy in men (hazard ratio, 1.81; 95% CI, 1.43–2.30) and women (hazard ratio, 2.11; 95% CI, 1.69–2.62). This led the authors to conclude that Hcy and UA are strongly associated with SCAF [115].

### 4.4. Adrenergic Effect

In a trial of 37 patients with essential hypertension (EH) compared with 37 healthy controls, blood levels of noradrenaline and adrenaline were demonstrated to be significantly higher in the EH group. Furthermore, the left ventricular mass index (LVMI) was also significantly higher in the EH group. The authors concluded that high levels of Hcy are associated with increased adrenergic activity in EH patients [116]. In a case control study of 273 patients with EH and 103 normotensive controls, the use of angiotensin-converting enzyme (ACE) inhibitors and beta-blockers significantly decreased and hydrochlorothiazide significantly increased plasma Hcy levels. The authors speculated that this reduction in Hcy levels was due to the improvement of endothelial function along with improved renal function [117]. In another trial of 120 patients with newly diagnosed hypertension, 100 mg of metoprolol per day was demonstrated to significantly reduce plasma Hcy levels. Additionally, there was no relation between homocysteine level and blood pressure control [118].

## 5. Discussion

AF represents the most common arrhythmia worldwide and is associated with significant morbidity and mortality [8]. The prevalence of AF in adults is about 2–4% and a 2,3-fold rise is expected in the years to come as a result of extended longevity in the general population and intensifying search for undiagnosed and untreated AF. A complex interplay of triggers and risk factors has been described widely in the literature, shedding light on the pathophysiologic substrate of the disease. 

Arterial hypertension, diabetes mellitus, chronic kidney disease, heart failure, valvular heart disease, coronary artery disease, inflammatory diseases, obstructive sleep apnea, chronic obstructive pulmonary disease, obesity, alcohol consumption, and smoking are only some of the risk factors implicated in the pathophysiology of AF. Thus it is evident that due to the complexity of the pathophysiologic substrate of the disease and the wide array of AF triggers, one individual biomarker is difficult to show adequate sensitivity and specificity in the prognostication of AF recurrences.

But why do we need a reliable biomarker for AF prognosis? First, AF diagnosis may be challenging due to asymptomatic and paroxysmal presentation; second, biomarkers could refine screening procedures, and third, they could help predict the risk of recurrent AF. Which is the profile of an ideal biomarker? It would be easily accessible, cost-effective, and demonstrative of consistent accuracy and reproducibility. In the present manuscript, we provide sufficient evidence connecting high levels of Hcy with AF paroxysms, through clinical research data and 2 convincing meta-analyses. Additionally, Hcy is an easily accessible biomarker with good reproducibility. In a recent paper by Chua et al. [119], a series of 40 different plasma biomarkers were validated by using logistic regression models and machine learning technologies; in this specific study, Hcy was not validated, while BNP and FGF-23 proved to hold the strongest association with AF. Interestingly, in the present scoping review we demonstrate evidence supportive of the strong connection between Hcy and BNP or NT-pro-BNP levels, which reinforces our belief that Hcy may serve in the prognostication of AF.

Inflammation and evolving fibrosis are thought to be the main pathophysiologic mechanisms responsible for the development of AF. A wide array of biomarkers have been associated with this process and have been already validated in the literature, as previously described. Which one is the best to provide the most accurate prognostic information, is still unclear. In the present manuscript, we tried to explore in depth what Hcy levels have to offer in this direction. Serious clinical data and meta-analyses, together with evidence connecting Hcy with other inflammation markers and oxidative stress, may confer adequate information. Additionally, it is well established that standard predictors of AF, such as left atrial volume, left atrial mechanical function, and left ventricular ejection fraction as evaluated by echocardiography, are strongly associated with fibrosis. Furthermore, left atrial fibrosis demonstrated by cardiac magnetic resonance imaging (CMR) has been strongly associated with AF recurrence. Since Hcy stands as a marker of cardiac inflammation, it might reflect the aforementioned left heart geometrical changes. Indeed, we present data that connect Hcy levels with LV mass hypertrophy and LV dilation. 

In our scoping review, we present data that implicate a direct adrenergic effect of Hcy in special populations, such as patients with arterial hypertension. This effect may cause left ventricular hypertrophy and promote left ventricular and left atrial remodeling, all of which are associated with AF development. By inhibiting sympathetic nerve drive by a b-blocker, one can speculate that this agent may inhibit the remodeling processes. B-blockers have been widely used for AF prevention and in the present manuscript, we provide evidence of Hcy levels reduction by metoprolol. Thus, we can propose that the beneficial effect of metoprolol in AF populations might be due to a bidirectional effect of b-blockade and Hcy levels reduction.

What about electrical remodeling and Hcy levels? ECG is the simplified mirror of the electrical circuits of the heart. Whether there is any convincing evidence that Hcy is associated with ECG changes that may serve as prognostic indicators of AF recurrences is still to be proven; there are reports pointing out that even in sinus rhythm, ECG might be a reliable biomarker of future AF events, since advanced interatrial block expressed by a p-wave duration > 120 ms and biphasic p-wave pattern in inferior leads, have been correlated with left atrial dilation [120,121]. Unfortunately, we were unable to find literature connecting Hcy levels with evidence of interatrial block or left atrial dilation. On the other hand, there is enough evidence of some association of Hcy levels with QRS duration with an undetermined effect on AF prevalence or recurrence. 

Machine learning applications in clinical cardiology have rapidly evolved in recent years. By using machine learning tools together with vast data sources, the management of a variety of chronic cardiac diseases including AF, is expected to change in the near future. The role of Hcy levels in this background remains unclear; large-scale trials are needed in order to establish which plasma AF biomarker is the most accurate to be incorporated in machine learning applications [122].

## 6. Future Research 

Our systematic search of the literature shed light on a wide array of existing data concerning the potential role of Hcy in the modification of risk factors for AF development. More specifically, our search demonstrated a direct effect of high Hcy levels on the adrenergic system in various laboratory models, while we highlighted the rather limited clinical evidence which presented a potential Hcy-lowering effect of beta-blockers. This potential for a dual benefit warrants further investigation in the clinical setting. 

Furthermore, we were able to identify several different biomarkers that could potentially be part of a predictive algorithm. Glutathione peroxidase is an endogenous antioxidant that may be inversely correlated to Hcy levels, while NT-pro-BNP and troponin levels also seem to hold a significant role. As NT-pro-BNP levels are mostly used in the diagnosis and monitoring of heart failure, this prohormone has been used in other disease states, such as myocardial ischemia [123]. Finally, UA has also been implicated as a predictor of future PAF events. While there does not seem to be one single biomarker that has been proven to consistently and accurately predict PAF, it seems very likely that a combination of these biomarkers may provide a valuable algorithm in that direction. Future research should be focused on combining the predictive value of these biomarkers to generate a reliable stratification algorithm.

## 7. Conclusions

In the context of PAF, there is sufficient evidence to support the use of Hcy levels as an independent predictor of future events. However, the pathogenesis of cardiac arrhythmias involves complex biological mechanisms that cannot be explained by or attributed to a single biomarker. Clinicians should take several other factors into consideration when assessing the likelihood of AF. In the near future, artificial intelligence with machine learning modalities could allow better prognostication and improved therapeutic management of the disease.

## Figures and Tables

**Table 1:** Reports indicating a positive correlation between levels of Hcy and future paroxysms of AF.

Reference	Number of Participants	Results
Kubota et al., 2019 [11]	7133 patients from Atherosclerosis Risk in Communities (ARIC) Study and Multi-Ethnic Study of Atherosclerosis (MESA)	An age-, sex-, and race-adjusted model showed dose-response relations between plasma Hcy concentrations and AF incidence in both ARIC and MESA studies A meta-analysis of both studies showed a significant association between homocysteine and AF Hcy may be a novel risk marker for AF.
Marcucci et al., 2004 [12]	310 NVAF patients on oral anticoagulant treatment (168 patients with previous ischemic events and 142 without) and 310 controls	Hyperhomocysteinemia was independently associated with NVAF after multivariate analysis A significant correlation was found between Hcy levels and LA diameter As shown by multivariate analysis, elevated Hcy levels were an independent risk factor for ischemic complications during NVAF.
Nasso et al., 2013 [13]	104 patients after minimally invasive epicardial ablation	Elevated circulating Hcy level, persistent type of AF, and increased LA dimension independently predicted the recurrence of AF during the follow-up Patients with a high Hcy level were more likely to have AF recurrence The cutoff value for elevated Hcy (16 μmol/L) yielded good diagnostic performance in the prediction of AF recurrence.
Naji et al., 2010 [14]	83 patients with persistent AF after successful electrical cardioversion	Patients were divided into 2 groups using a cut-off value for the last quartile of plasma Hcy concentration (>14.4 μmol/L) Kaplan Meier analysis showed a statistically significant difference in AF recurrence rates between both groups after 18 months Predictors of AF recurrence were the duration of AF, treatment with amiodarone, and Hcy level < or =14.4 μmol/L Hcy levels determined prior to electrical cardioversion can predict recurrence of AF after successful restoration of sinus rhythm.
Shi et al., 2016 [15]	132 patients with both hypertension and AF (78 with paroxysmal AF and 84 with persistent AF) and 136 hypertensive patients	Compared with paroxysmal AF patients, persistent AF patients had higher serum Hcy concentration and larger LA diameters In patients with hypertension, the presence of AF was associated with arterial stiffness; serum Hcy levels may reflect mechanisms behind this association.
Shimano et al., 2008 [16]	62 paroxysmal or persistent AF patients undergoing RFCA	Plasma Hcy levels were significantly higher in patients with persistent AF compared with levels in paroxysmal AF and control patients Hcy levels positively correlated with LA dimension While no significant correlation was found between basal Hcy levels and recurrent AF after RFCA in AF patients, patients in the high Hcy group exhibited a significantly higher rate of cardiovascular events without AF recurrence compared with those in the low Hcy group.
Schnabel et al., 2010 [17]	3120 Framingham cohort participants	10 biomarkers representing inflammation (CRP, fibrinogen, BNP, NT-pro BNP, Hcy, renin, aldosterone, D-dimer, plasminogen-activator inhibitor 1, urine-albumin excretion) were associated with incident AF over a median follow-up of 9.7 years In multivariable-adjusted analyses, the biomarker panel was associated with incident AF.
Cingozbay et al., 2002 [18]	38 patients with non-valvular AF divided into two groups: group A (patients with AF and stroke) and group B (AF without stroke) plus a reference group of 15 patients	Group A had a statistically higher Hcy level not only than group B, but also than the reference group While 60% of group A (*n* = 12) had the elevated Hcy level, the rate was only 22% for group B (*n* = 4) Hyperhomocysteinemia may be one of the explanations for the increased rate of thromboembolic complications in older patients with AF.
Yao et al., 2017 [19]	257 consecutive patients with persistent AF who underwent catheter ablation	Plasma Hcy levels were significantly elevated in patients with early recurrence compared with those without early recurrence In multivariate analysis, Hcy was significantly associated with early recurrences The optimal cut-off value was 14 µmol/L for Hcy Patients with Hcy ≥ 14 µmol/L had a higher early recurrence rate compared with those with Hcy <14 µmol/L.
Yao et al., 2017 [20]	Review	Evidence has well documented the close relationships between Hcy and AF Hcy plays an important role in a number of vascular diseases having a strong association with AF. The possible mechanisms linking elevated Hcy and cardiovascular events in AF patients include oxidative stress, inflammatory response and atrial remodeling.
Giusti et al., 2007 [21]	456 NVAF patients and 912 matched controls	Hcy was higher in patients than in controls In both populations, a genotype-phenotype association between Hcy and C677T MTHFR polymorphism was observed In controls, a significant (*p* = 0.029) association between tHcy and −786C/T eNOS polymorphism was also observed At the multivariate analysis, the NVAF risk significantly increased in the upper quartiles of Hcy compared to the lowest.
Svenningsson et al., 2020 [22]	3535 patients with no history of AF	Higher plasma Hcy and were associated with increased risk of incident AF.
META-ANALYSES		
Rong et al., 2020 [23]	11 studies with 3974 patients	Compared with control subjects, Hcy levels were higher in PAF and persistent AF patients Persistent AF patients had a higher level of Hcy compared with PAF patients The pooled analysis indicated that AF patients with recurrence had significantly higher Hcy levels than those without recurrence.
Dong et al., 2021 [24]	5 studies with 13,556 patients	The serum or plasma Hcy levels were significantly associated with AF Sensitivity analysis showed that the main results remained unchanged after omitting any single study or converting the random effects model to a fixed effects model.

Abbreviations: Hcy: homocysteine, AF: atrial fibrillation, NVAF: non-valvular atrial fibrillation, LA: left atrial, RFCA: radiofrequency catheter ablation, CRP: C-reactive protein, BNP: brain natriuretic peptide, PAF: paroxysmal atrial fibrillation.

**Table 2:** Table presenting the outline of studies included in the scoping review.

Reference	Model	Potential Mechanism
Bayrak et al., 2021 [25]	Copenhagen rats	Oxidative Stress
Borkowska et al., 2021 [26]	HUVEC and SH-SY5Y cells	Oxidative Stress
Cheng et al., 2021 [27]	C57BL/6 mouse aortae ex vivo	Oxidative Stress
Guo et al., 2021 [28]	24 studies assessed	Oxidative Stress
Sharma et al., 2021 [29]	several proteins and enzymes.	Oxidative Stress
Boyacioglu et al., 2014 [30]	Wistar rats	Oxidative Stress
Aminzadeh et al., 2018 [31]	H9C2 myocardial cells	Oxidative Stress
Derouiche et al., 2014 [32]	Male Wistar rats (Pasteur Institute-Algiers)	Oxidative Stress
Aissa et al., 2017 [33]	Mice	Oxidative Stress
Dittoe et al., 2011 [34]	Rat neonatal cardiomyoblasts (H9c2 cells)	Oxidative Stress
Kolling et al., 2011 [35]	Hearts of young rats	Oxidative Stress
Devi et al., 2006 [36]	Spontaneously hypertensive rats	Oxidative Stress
Han et al., 2020 [37]	C57BL/6J mice	Oxidative Stress
Mendes et al., 2010 [38]	Male Wistar rats	Oxidative Stress
Singh et al., 2008 [39]	Rats	Oxidative Stress
Stojanovic et al., 2016 [40]	Isolated rat hearts	Oxidative Stress
Timkova et al., 2016 [41]	Rats	Oxidative Stress
Yalçinkaya-Demirsözm et al., 2009 [42]	Rabbits	Oxidative Stress
Chang et al., 2004 [43]	Rat myocardial mitochondria	Oxidative Stress and Taurine
Chang et al., 2004 [44]	Rat isolated myocardial mitochondria	Oxidative Stress and Taurine
Chang et al., 2008 [45]	Rats	Oxidative Stress and H2S
Wang et al., 2015 [46]	Rats	Oxidative Stress and H2S
Givvimani et al., 2011 [47]	Mouse cardiac endothelial cells	Oxidative Stress and Fibrosis
Joseph et al., 2008 [48]	Rat model	Oxidative Stress and Fibrosis
Li et al., 2017 [49]	Six-week-old C57BL6/J mice	Oxidative Stress and Fibrosis
Tyagi et al., 2005 [50]	Mice	Oxidative Stress and Fibrosis
Shi et al., 2021 [51]	2–3 days old Wistar rats	Fibrosis
Zhao et al., 2021 [52]	C57BL/6 mice with a high L-methionine (L-MET) diet for 12 weeks	Fibrosis
Carroll et al., 2005 [53]	Rabbit model	Fibrosis
Zulli et al., 2006 [54]	Rabbits	Fibrosis
Han et al., 2020 [55]	Left atrial appendage from patients with either sinus rhythm (SR) or AF	Fibrosis
Zhi et al., 2013 [56]	Mice	Fibrosis
Zhang et al., 2016 [57]	Apolipoprotein E-deficient (ApoE −/−) mice and neonatal rat cardiac fibroblasts (CFs)	Fibrosis
Muthuramu et al., 2015 [58]	Female C57BL/6 low-density lipoprotein receptor (Ldlr (−/−)) cystathionine-β-synthase (Cbs (+/−)) mice	Fibrosis
Wang et al., 2016 [59]	Cardiocytes H9C2	Fibrosis
Chaouad et al., 2019 [60]	Sand rat Psammomys obesus	Fibrosis and Remodeling
Joseph et al., 2005 [61]	Mast cell-deficient rat model	Fibrosis, Remodeling and Diastolic Dysfunction
Joseph et al., 2004 [62]	Hypertensive rats	Fibrosis, Remodeling and Diastolic Dysfunction
Cao et al., 2021 [63]	Hypertensive rats	Fibrosis and Diastolic Dysfunction
Li et al., 2021 [64]	Mouse CFs	Fibrosis and Diastolic Dysfunction
Cao et al., 2021 [63]	Wistar Kyoto (WKY) and spontaneous hypertension rats (SHR)	Remodeling
Chaturvedi et al., 2014 [65]	HL-1 cardiomyocytes and mouse models (CBS+/−)	Remodeling
Herrmann et al., 2007 [66]	Rats	Remodeling
Jeremic et al., 2018 [67]	Adult male Wistar albino rats	Remodeling
Kar et al., 2019 [68]	Male CBS(+/−) and sibling CBS(+/+) (WT) mice	Remodeling
Raaf et al., 2011 [69]	Rats	Remodeling
Mishra et al., 2009 [70]	HL-1 cardiomyocytes	Remodeling
Rosenberger et al., 2011 [71]	male C57/BL6J mice	Remodeling
Li et al., 2021 [64]	Male C57BL/6J mice	Endothelial dysfunction
Ables et al., 2015 [72]	Μice	ECG
Cainzos-Achirica et al., 2021 [73]	1407 participants (61% women) without diabetes or severe hypercholesterolemia	Calcium
Cheng et al., 2021 [27]	Human umbilical vein endothelial cells (HUVECs)-derived EA.hy926 immortalized cells	Calcium
Cai et al., 2011 [74]	Wistar rat hearts	Calcium
Shontz et al., 2001 [75]	Whole-cell voltage-clamp recordings were made in ventricular myocytes isolated from normal rat hearts	Ca2+-independent, transient outward Potassium (K+) current (I(to))
Sun et al., 2021 [76]	Residual internal mammary artery (IMA) segments obtained from patients undergoing CABG	Potassium Calcium (K(Ca))
Cai et al., 2007 [77]	Human atrial cells	Potassium
Lopatina et al., 2015 [78]	Chicken embryo cardiac tissue explants	Potassium, Sodium
Cai et al., 2009 [79]	Human atrial monocytes.	Sodium
Pacher et al., 1999 [80]	Isolated rat hearts	Sodium
Soni et al., 2016 [81]	Wild-type mice (WT)	Magnesium
Han et al., 2020 [82]	Mouse atrial myocytes (MACs) obtained from C57B6 mice.	Electrical Remodeling
Mishra et al., 2011 [83]	cardiomyocytes obtained from C57BL/6J (WT) and db/db mice.	β2-AR
Mishra et al., 2010 [84]	12 week male diabetic Ins2+/− Akita and C57BL/6J mice	β2-AR
Tasatargil et al., 2006 [85]	Adult male Wistar rats	β2-AR
Moshal et al., 2009 [86]	Cardiomyocyte-specific knockout of NMDA-R1	NMDA-R1
Moshal et al., 2008 [87]	C57BL/6J mice	NMDA-R1
Tyagi et al., 2010 [88]	Cardiac-specific knockout (KO) of NMDA-R1	NMDA-R1
Srejovic et al., 2017 [89]	Hearts of Wistar albino rats	NMDA-R1
Busingye et al., 2021 [90]	600 human population	Inflammation
Ji et al., 2020 [91]	human umbilical vein endothelial cells (HUVECs)	Inflammation
Xie et al., 2021 [92]	mice	Inflammation

**Table 3:** Studies reporting available clinical data regarding the connection between Hcy and AF.

Reference	Number of Participants	Results
Schnabel et al., 2005 [102]	643 patients with coronary artery disease	Hcy and was among the strongest univariate predictors of future cardiovascular risk, even after adjustment for cardiovascular confounders Hcy levels were significantly elevated in individuals with future cardiovascular events.
Ay et al., 2003 [103]	42 consecutive patients with ischemic stroke caused by nonvalvular AF	Mean Hcy levels were significantly higher in patients with LA thrombus Multivariate logistic regression analysis showed that the effect of high Hcy was independent of other clinical or echocardiographic variables known to increase LA thrombus.
Loffredo et al., 2005 [104]	163 consecutive patients with permanent (*n* = 118) or paroxysmal (*n* = 45) AF of non-valvular origin hospitalized for cardiac reasons	Multivariate analysis showed that total Hcy and fibrinogen were independently associated with ischemic stroke With respect to patients in the first quartile of the Hcy distribution (4.6–7.5 μmol/L), patients in the fourth quartile of the Hcy distribution (18.7–67.1 μmol/L) had a 2.73-fold increased probability of ischemic stroke.
Sundström et al., 2004 [105]	2697 Framingham Heart Study participants free of heart failure and previous myocardial infarction	Plasma Hcy was positively related to LV mass, wall thickness, and relative wall thickness in women, but not in men.
Alter et al., 2010 [106]	66 individuals with suspected cardiomyopathy	Hyperhomocysteinemia (>12 μmol/L) was found in 45 patients (68%) LV mass was greater in these patients compared with individuals with normal Hcy Hcy was increased in patients with increased brain natriuretic peptide LV mass, LV end-diastolic and end-systolic volume were significantly increased in individuals in the upper quartile compared with the lower quartile (90 +/− 25 vs. 65 +/− 18 g/m^2^, *p* = 0.021; 114 +/− 50 vs. 71 +/− 23 mL/m^2^, *p* = 0.042; 76 +/− 51 vs. 36 +/− 22 mL/m^2^, *p* = 0.045) LV dilatation was more common in hyperhomocysteinemia (>12 μmol/L, *p* = 0.0166).
Li et al., 2017 [107]	7002 healthy individuals	The distribution of Hcy levels was determined for an entire population after the data were grouped into quartiles (Q1: ≤11.1 μmol/L; Q2: 11.1–13.8 μmol/L; Q3: 13.8–18.2 μmol/L; Q4: >18.2 μmol/L) The mean value of the QTc interval in each quartile was 433.2 ± 23.8 ms, 430.0 ± 24.6 ms, 429.2 ± 24.5 ms and 430.6 ± 25.7 ms Multiple logistic regression analyses showed that, compared with the second quartile, and after fully adjusting for potential confounding factors, the odds for QTc > 440 ms in the first and fourth quartile increased (*p* < 0.05), (OR: 1.23, 95% CI: 1.05–1.43 for Q1; OR: 1.40, 95% CI: 1.19–1.65 for Q4).
Leng et al., 2015 [108]	178 healthy individuals	Mean population Hcy plasma levels were 10.4 μmol/L (SD = 3.6) The mean QRS duration was 101.8 ms (SD = 17.4) Groups were stratified on the basis of QRS duration (≤120 ms [*n* = 157] and >120 ms [*n* = 21]) QRS duration subgroup (≤120 ms vs. >120 ms) mean differences across Hcy levels were 10.1 μmol/L (SD = 3.3) and 12.2 μmol/L (SD = 4.7), respectively (*p* = 0.016) Other ECG parameters (PQ interval, QTc interval, and QT dispersion) measurements were not significantly associated with differences in plasma Hcy.
Guéant-Rodriguez et al., 2007 [109]	515 patients with coronary artery disease and 194 patients without evidence of coronary artery lesion	Hcy levels were significantly higher in the 187 patients with a low LVEF (<40%) than in those without ventricular dysfunction LVEF, NYHA functional class II or III and coronary artery disease, stable angina, and hypertension were clinical characteristics that influenced total Hcy level in univariate analysis Hcy was significantly associated with LVEF and NT-pro-BNP in univariate regression and in multiple regression LVEF was a predictor of homocysteine >15 μmol/L in the whole population and in patients without documented coronary artery disease.
Guéant-Rodriguez et al., 2013 [110]	1020 subjects including patients undergoing coronarography and ambulatory elderly subjects	Folate deficit was more frequent in the coronarography population than in the elderly ambulatory volunteers and produced a higher concentration of Hcy Subjects with Hcy in the upper quartile (≥18 μmol/L) had higher concentrations of NT-pro-BNP compared to those in the lower quartile (≤12 μmol/L), in both populations Hcy and NT-pro-BNP were positively correlated with short chain-, medium chain-, long chain-acylcarnitines and with acylcarnitine ratios indicative of decreased mitochondrial acyldehydrogenase activities In multivariate analysis, homocysteine and long-chain acylcarnitines were two interacting determinants of NT-pro-BNP, in addition to LVEF, BMI, creatinine, and folate.
Görmüş et al., 2010 [111]	31 patients with type 2 diabetes mellitus	Plasma Hcy levels were significantly higher in diabetics than in controls Positive correlation was noted between NT-proBNP and Hcy levels in diabetic patients with left ventricular dysfunction.
Cho et al., 2006 [112]	227 patients with cardiovascular disease	Patients homozygous for the TT mutation had the highest plasma Hcy levels compared with wild-type CC homozygotes and CT mutant heterozygotes Plasma BNP concentrations were significantly higher in patients with MTHFR C677T mutation compared to patients without the mutation Plasma BNP concentrations were positively correlated with Hcy concentrations Multivariate logistic regression analysis showed that elevated concentrations of BNP, CRP, Hcy, and the presence of the MTHFR C677T mutation independently contributed to the prediction of cardiovascular diseases.
Ye et al., 2014 [113]	1497 healthy individuals	Serum Hcy was associated with a higher likelihood of detectable hs-cTnT A subsequent subgroup analysis found that in subjects aged 65 years and older, the association between hs-cTnT levels and Hcy levels was strengthened.
Alam et al., 2012 [114]	194 consecutive patients with acute myocardial infarction	The mean (+/−SD) serum Hcy level was 20.2 +/− 14.3 μmol/L with a range from 7.4 to 129.1 μmol/L Mean serum troponin-I level was classified according to normal (<15 μmol/L) and high (> or =15 μmol/L) levels of serum Hcy values The mean serum troponin-I level was 8.9 +/− 8.6 ng/mL in the patients having normal serum Hcy level and 18.4 +/− 6.5 ng/mL in the patients having high serum Hcy level A significant positive correlation was found between serum troponin-I level with Hcy level Patients with moderate hyperhomocysteinemia (> or =15 μmol/L) were found to be 7.09 times more likely to have increased serum troponin-I (a surrogate marker of the extent of the myocardial injury).
Wang et al., 2022 [115]	1224 consecutive patients with cardiac implantable electronic devices	On multivariable Cox regression analysis with potential confounders, elevated Hcy and UA biomarkers were significantly associated with an increased risk of subclinical AF A rise of 1 SD in Hcy (5.7 μmol/L) was associated with an increased risk of subclinical AF in men and women regardless of their UA levels Similarly, a 1-SD increase in UA (91 μmol/L) was associated with an increased risk of subclinical AF among the patients with high levels of Hcy in men (hazard ratio, 1.81; 95% CI, 1.43–2.30) and women (hazard ratio, 2.11; 95% CI, 1.69–2.62).
Wocial et al., 2002 [116]	37 patients with mild essential hypertension (EH) and 37 healthy volunteers	Hcy was significantly higher in patients with EH (8.7 +/− 2.4 vs. 6.6 +/− 1.3 μmol/L; *p* < 0.01).
Poduri et al., 2008 [117]	273 patients with essential hypertension (EH) and 103 normotensive controls	ACE inhibitors and beta-blockers significantly decreased and hydrochlorothiazides significantly increased the plasma Hcy levels in hypertensive patients No significant association between MTHFR C677T genotypes and changes in Hcy levels in response to antihypertensive was observed in EH patients.
Atar et al., 2005 [118]	120 patients with newly diagnosed hypertension	Hcy levels decreased significantly by the end of the 4th month when compared with basal values There was no relation between Hcy level and blood pressure control There was a significant decrease in Hcy levels in the women treated in this study; however, this effect was absent in men.

Abbreviations: Hcy: homocysteine, AF: atrial fibrillation, LA: left atrial, LV: left ventricular, ECG: electrocardiogram, LVEF: left ventricular ejection fraction, NYHA: New York Heart Association, BMI: body mass index, BNP: brain natriuretic peptide, CRP: C-reactive protein, hs-cTnT: high sensitivity cardiac troponin T, SD: standard deviation, UA: uric acid, ACE: angiotensin-converting enzyme, EH: essential hypertension.

## Supplementary Materials

The following supporting information can be downloaded at: https://www.mdpi.com/article/10.3390/diagnostics12092192/s1, Table S1: Preferred Reporting Items for Systematic reviews and Meta-Analyses extension for Scoping Reviews (PRISMA-ScR) Checklist. Reference [124] is cited in Supplementary Materials.
